# Transactions of the Buffalo Obstetrical Society

**Published:** 1885-03

**Authors:** 


### TRANSACTIONS OF THE BUFFALO OBSTETRICAL SOCIETY.

*Stated Meeting, Jan. 27, 1885.*

The President, Dr. W. W. Potter, in the Chair.

The subject for discussion was announced as, "Is there a Kinship in the Infection of Puerperal Fever, Erysipelas and Sarlatina?"

Dr. W. S. Tremaine, in opening the discussion, said that in regard to the nature of so-called puerperal fever, there were two schools which had been characterized as the "Essentialists" and the "Absorptionists." He thought it was always well to have a definite idea, if possible, as to the nature of the morbid process to which we give a distinct name. For his part, he believed there was ground for reconciliation between the opposing views —perhaps, even, both were right.

There was, he said, a form of septicæmia to which puerperal women were subject, as, indeed, were pregnant, and even non-pregnant women, accompanied, of course, by that rise of temperature ordinarily called "fever," but which did not differ materially from septicæmia occurring from wounds in either males or females. On the other hand, cases of fever of a continued, or slightly remittent type, were seen in puerperal women by physicians of any extended experience, which were not apparently caused by any septic poison, unless all varieties of fever were, indeed, due to some forms of septic infection, which latter would seem not an impossible view to take, if the modern teachings of some pathologists as to the potency for evil of the different microbes which they describe was true.

But, he continued, the nature of fever in general, and the different theories in regard to its causation, was too extensive a subject to dwell upon here. To return, therefore, to that particular species usually designated as "puerperal fever," he believed that puerperal septicæmia would be a better term for it. There seemed little room for doubt that it was due to a *contagium.*

He presumed the real question for discussion at this time was: Is that contagium identical with that of erysipelas and scarlet fever, or, is there any distinct relation between them?

From a clinical standpoint there were cases which seemed to prove their common origin; while, on the other hand, there were facts which appeared to conclusively contradict this view. There were so many factors to which it was difficult to give a determinate value, that the solution of the question could, for the present, at least, be, perforce, only an approximative one, and, as it was obviously impossible to carry on, carefully and *intentionally*, inoculation experiments in puerperal women, the question must be determined, if determined at all, by inference from the behavior of other well-known varieties of *contagia*, and also from a study of the clinical history of the disease.

He had seen one case of well-marked *pyæmia* in a puerperal woman, at a dry, elevated point, isolated, where no other case had ever happened, and where, certainly, there had never been any cases of erysipelas or scarlet fever. This disease rarely occurred sporadically; it was generally epidemic, or endemic, in a particular locality. There was, unquestionably, at times a greater tendency to all these forms of disease than at others, either from a higher concentration of the *contagium*, or from an increased susceptibility. But vital statistics did not show that during epidemics of scarlet fever there were more cases of puerperal septicæmia.

He did not, in conclusion, for his own part, think that the identity of the poisons was yet proven. Why not, said he, include diphtheria? for he had repeatedly seen a diphtheritic membrane on the gentalia of women suffering from so-called puerperal fever.

Dr. M. Hartwig said that it was difficult to arrive at an absolute understanding of the etiology of puerperal fever, because of the inadmissibility of experimental reproductions of the same in man, but observation and experiment in regard to putrefaction, and the intrduction of this process into the animal economy, had shed much recent light on the subject. His personal convictions were in favor of the Fordyce Barker idea, viz., that there are two forms of puerperal fever, one septic or putrid, the other a disease, *sui generis*, probably possessing a peculiar germ of its own. The first, or putrid form, comprises the vast majority of cases, and can originate by the introduction of putrid matter from without, as well as from the body itself. It manifests itself according to the several ways in which the poison courses within the system, as parametritis, perimetritis (*sive* local peritonitis) then general peritonitis, lymphangitis and venous metastatic pyæmia. Though he was formerly doubtful as to putrefaction ever originating within the body—*i. e.*, whether so-called self-infection exists or ever occurs—he now does not question the fact. Billroth's and Zeller's researches show that the bacteria of putrefaction exist constantly in the blood, and probably within the tissues of the living body; and further, that they do not have an opportunity for development generally, because either the oxygen or ozone of the blood destroys them incessantly, or because the living cells, fighting for their own life, do not give them a chance to develop. Only peculiar circumstances—a weakened condition of the human frame—afford them, occasionally, an opportunity to start into a violent activity.

Dropping theory, for the present, he asserted that practice had exhibited the fact to him that self-infection—at least purulent if not putrid processes—does occur in the body, though seldom spontaneously, except from the introduction of a germ from without. That putrefaction, without germs, does not occur, we know, from the fact that goods properly canned never spoil.

He then related several examples of purulent inflammation without an external lesion or outer introitus for the entrance of germs,and cited his personal experience to show how rarely putrefactive changes occur spontaneously, for in twelve years of practice he had not witnessed a single case. He had dwelt broadly upon this subject, for only through an understanding of putrefactive changes in general can the true clue to the proper view of puerperal fever be obtained. Now, said he, in the womb we have a surface communicating, often, only too readily with the outside world, and thus germs might be introduced without especial occasion for such introduction at the time of child-birth, and even without examination on the part of the attendant. A seemingly spontaneous puerperal fever can undoubtedly occur, though it must be rare, for, as a rule, the uterus escapes the introduction of germs from without, especially the germs of putrefaction, unless they are brought into its cavity by manipulation during labor. He cited two examples illustrating this point. Once he was attending a case of abortion where he purposely abstained, totally, from digital examination of the genitalia for eight days of constant flowing. At last it grew too violent, the discharges began to smell, and he evacuated the uterus with the curette. About one-half of the mass was in the vagina, the other half in the uterus. The latter was in a normal condition with regard to putrefactive changes, but the vaginal part was decayed. At another time, lately, he left one-half the membranes in utero, after a timely birth. His antiseptic precautions were perfect. Four days later the membranes came with colicy pains, not putrid in the least.

In conclusion, he thought these facts, as well as others which he had not time now to cite, went to show that the opinion which he expressed at the outset was correct, viz., that puerperal fever is, as a rule, a putrid change introduced from without, and proper antisepsis is the guard against it. Self-infection from the uterus and vagina, and infection through other channels of entrance, like the lungs, the skin, the intestines, etc., are rare and not of practical consideration now, but they seemingly do occur occasionally, and must not be altogether overlooked in the general discussion of the subject. He thought he had seen one case of fulminant puerperal fever, without putrescent or inflammatory changes in or around the uterus, which he took to be the puerperal fever, *sui generis* (Barker's).

Such cases might have something to do with erysipelas or scarlet fever, or might have a germ of their own. As a rule he did not think handling an erysipelas or scarlet fever patient prevents attending a confinement on the same day, if proper antisepsis is used. He had seen, only once, a mother with parametritis while the child had erysipelas, but the child's affection was later. On the other hand, he had seen several normal puerperia where scarlet fever existed in the same room. Modern research, especially Koch's, shows conclusively that each infectious disease has a specific germ of its own, and thus we may get, in the future, besides the germs of putrefaction and ordinary puerperal fever, one of the puerperal fever *sui generis.*

Dr. S. Y. Howell said that he believed mainly in the views expressed by Dr. Hartwig, and related the history of a case showing the connection between malignant scarlet fever and puerperal fever, and where, unquestionably, scarlatinal poison caused the puerperal septicæmia.

Dr. P. W. Van Peyma said that in one of the hospitals, at the time he was a resident there, a number of cases of erysipelas were in the surgical wards at the same time that puerperal fever occurred in the lying-in ward. There was, of course, some communication between the wards, though the attendants were, as a rule, limited to their respective wards. Still there may have been inoculation. At one time it became necessary to close up the lying-in ward for a number of weeks. Similar experiences had occurred in other hospitals, as, for instance, in Bellevue, when he was there with Dr. Lusk, when it was the opinion that the original cause was erysipelas. It seemed to him quite important to recognize the fact that a puerperal woman may also have any disease that any other woman may have. It was quite important to always empty the uterus thoroughly at the time of labor. He had had very few--he did not remember a single instance—of puerperal fever in women whom he had attended from the beginning. He had often seen it in cases attended by midwives, who are very careless in leaving portions of the placenta behind.

Dr. Thomas Lothrop believed that we have a puerperal fever, which is a distinct entity, though the great majority of cases are antogenetic. There are cases, however, which cannot be traced to any such cause. The lochia will be perfectly free from putridity, and, as far as we can observe, there is no avenue for the absorption of septic material, and yet a violent form of puerperal fever is present. If it was not a disease, *sui generis*, he did not know what to call it. He believed, however, that the greater number of cases were antogenetic—distinctly septic in origin. As to the relationship between puerperal fever, erysipelas and scarlet fever, he thought that great care should be exercised in attending puerperal women when these cases are also under observation, at the same time. He could not say that he had ever seen puerperal fever arise from any such source, but he had always been afraid of it, and thought that practitioners who are treating zymotic diseases should be exceedingly careful in their relations to puerperal women.

Dr. C. C. Frederick's practical experience had been limited, but he had discovered a great diversity of opinion among medical men on the subject, and thought that the truth must be found between the two extremes. He thought he had seen cases that were undoubtedly due to septic infection, and he was equally sure that he had seen cases where there was no discernible cause of the disease. The first case he ever saw, he thought he communicated to the patient himself, while he was yet a student. He went directly from the dissecting-room, only washing his hands with warm water and soap, and attended her. Four or five days afterwards she developed metritis, perimetritis, etc., and came very near dying. It seemed to him that, in reading of the epidemics in different places, there must be a puerperal fever developed from a specific germ.

Dr. R. L. Banta thought that we were in the dawn of discovery in this matter, though discussions, thus far, had not given us great light. Nobody yet seemed fully satisfied, no matter what theory was advanced or advocated. He related the case of a physician where the poison, or infectious material, lurked about him for a number of weeks, when he finally abandoned obstetric practice to get rid of the apparently strong suspicion that such was the fact. During the last ten or twelve months he himself had been using corrosive sublimate water, washing his hands in it freely every time he approached a puerperal woman, which he thought was a safe plan, to say the least. About two months ago he had two children (his own) sick with diphtheria, which he believed was one of the causes said to produce puerperal fever. His wife had diphtheria, and he, himself, had a mild attack, during which he attended a woman in labor, being careful to use the sublimate solution, and she had no signs of puerperal fever.

Dr. C. G. Stockton said that the subject seemed to be shrouded in mystery, but he believed it to be a zymotic disease in the majority of cases. Dr. Tremaine had given us a very interesting instance of a case on the plains where there could be no possible connection with other cases, which reminded him of a case of scarlet fever which was reported a year ago, by a medical gentleman residing in Dakota. The family, living secluded from other people, fell sick with scarlet fever, and the physician reported it as an interesting instance, where the disease had arisen without contact with the outer world. We always hear of such instances as related by Dr. Banta. One of his earliest recollections is of a similar fact occurring in the practice of his own father, and which followed him for nearly a year.

Dr. Wm. B. Hawkins said that it would seem natural for any one, who had seen cases they were sure were due to sepsis, to accept the theory of septic infection for all cases. He had never seen any cases that did not seem explainable on that theory. When we considered the large ex ent of surface exposed in the puerperal state, and the great chances for absorption that exist, it seemed very easy for the disease to arise from anto-infection.

Dr. C. R. Jewett, said it seemed to him that puerperal fever never arises except from a septic source. He further believed that the site of the infection, in ninety cases out of one hundred, was before it reached the uterus, and resided either in the vagina or vulva. He once saw a mother nursing a child with an erysipelatous inflammation of the neck. This was after delivery, some sixteen or seventeen days, and he observed the case very carefully. The woman developed puerperal fever and died of it. He thought there was a great difference in the degree of the infection. In the convalescent ward of the Maternity Hospital in New York, where patients were taken when the uterus had undergone involution so it could no longer be felt above the pubes, he saw septic troubles occur, and the women were kept in the ward without affecting the other women there; but the nurses were not allowed to have anything to do with the other patients. There seemed to him no longer any doubt, in the minds of the best men, about the septic origin of puerperal fever.

Dr. Hartwig said he was glad to hear most of the gentlemen corroborate his opinion; that there were two distinct diseases that we call puerperal fever; one is carried through the air, and the other is in consequence of putrid matter in the region of the genitals. He believed the day would come when the germs of non-putrid fever will be cultivated. In regard to erysipelas, we know very definitely what causes it. The micrococcæ is already raised and cultivated, and it is an entity of itself. He wished to oppose Dr. Van Peyma. He did not think that leaving a few pieces of placenta behind does any harm if cleanliness is absolute.

Dr. Tremaine, in closing the discussion, said he would like to ask: Is not all fever septic? Is not fever evidence of a septic influence in the system? Is it possible that fever can occur without that? Is not fever always due to some poison, either produced in the system or injected from without? That puerperal fever, as generally understood, is of septic origin, goes without saying. The term puerperal fever is not specific enough. We should say puerperal metritis, peritonitis, lymphangitis, etc.

There was no such thing as disease as an entity of itself. Disease is disturbed normal action, by some disturbing cause, either intrinsic or extrinsic. If there is a direct relation between erysipelas and puerperal fever, if the germ of one can produce the other, why does not the contrary relation hold good? Has puerperal fever ever been shown to produce erysipelas? Unquestionably there are forms of fever, not recognized as septic from the present standpoint, that occur in the puerperal state. The puerperal state is exceedingly prone to febrile conditions; there is, we say, a great tendency to disturbance of the system, and if it be true, how shall we account for the hundreds and hundreds of thousands of women who have no trouble except a mechanical one? As regards the dissecting-room question, that has been pretty clearly settled. Dr. Sands, of New York, who was a demonstrator of anatomy, attended hundreds of cases and had no puerperal fever resultant. I would myself, however, hesitate to attend a woman in labor if also attending, at the same time, a case of erysipelas or scarlet fever, simply because there is a feeling, more or less widespread, that these diseases may produce puerperal fever. For his own part, he thought the germ theory of these diseases is the most logical one. There must be some cause for this production of zymotic disease, and it seemed plausible that the germ theory is in the right direction, though we are all the time surrounded with difficulty in its elucidation.

